# Response to Infliximab in Crohn's Disease: Genetic Analysis Supporting Expression Profile

**DOI:** 10.1155/2015/318207

**Published:** 2015-08-03

**Authors:** Luz María Medrano, Carlos Taxonera, Cristina González-Artacho, Virginia Pascual, María Gómez-García, Manuel Barreiro-de Acosta, José L. Pérez-Calle, Fernando Bermejo, Antonio López-Sanromán, Dolores Martín Arranz, Javier P. Gisbert, Juan Luis Mendoza, Javier Martín, Concepción Núñez, Elena Urcelay

**Affiliations:** ^1^Immunology Department, Hospital Clínico San Carlos, Instituto de Investigación Sanitaria del Hospital Clínico San Carlos (IdISSC), 28040 Madrid, Spain; ^2^Gastroenterology Department, Hospital Clínico San Carlos, Instituto de Investigación Sanitaria del Hospital Clínico San Carlos (IdISSC), 28040 Madrid, Spain; ^3^Gastroenterology Department, Virgen de las Nieves Hospital, 18014 Granada, Spain; ^4^Gastroenterology Department, Hospital Clínico Universitario de Santiago de Compostela, 15706 Santiago de Compostela, Spain; ^5^Gastroenterology Department, Alcorcón Hospital, 28922 Madrid, Spain; ^6^Gastroenterology Department, Fuenlabrada Hospital, 28942 Madrid, Spain; ^7^Gastroenterology Department, Ramón y Cajal Hospital, 28034 Madrid, Spain; ^8^Gastroenterology Department, La Paz Hospital, 28046 Madrid, Spain; ^9^Gastroenterology Department, Hospital Universitario La Princesa, Instituto de Investigación Sanitaria Princesa (IP), 28006 Madrid, Spain; ^10^Centro de Investigación Biomédica en Red de Enfermedades Hepáticas y Digestivas (CIBEREHD), 28002 Madrid, Spain; ^11^Instituto de Parasitología y Biomedicina López-Neyra, IPBLN-CSIC, Armilla, 18100 Granada, Spain

## Abstract

Substantial proportion of Crohn's disease (CD) patients shows no response or a limited response to treatment with infliximab (IFX) and to identify biomarkers of response would be of great clinical and economic benefit. The expression profile of five genes (*S100A8-S100A9, G0S2, TNFAIP6*, and* IL11*) reportedly predicted response to IFX and we aimed at investigating their etiologic role through genetic association analysis. Patients with active CD (350) who received at least three induction doses of IFX were included and classified according to IFX response. A tagging strategy was used to select genetic polymorphisms that cover the variability present in the chromosomal regions encoding the identified genes with altered expression. Following genotyping, differences between responders and nonresponders to IFX were observed in haplotypes of the studied regions:* S100A8*-*S100A9* (rs11205276^*^G/rs3014866^*^C/rs724781^*^C/rs3006488^*^A; *P* = 0.05);* G0S2* (rs4844486^*^A/rs1473683^*^T; *P* = 0.15);* TNFAIP6* (rs11677200^*^C/rs2342910^*^A/rs3755480^*^G/rs10432475^*^A; *P* = 0.10); and* IL11* (rs1126760^*^C/rs1042506^*^G; *P* = 0.07). These differences were amplified in patients with colonic and ileocolonic location for all but the *TNFAIP6* haplotype, which evidenced significant difference in ileal CD patients. Our results support the role of the reported expression signature as predictive of anti-TNF outcome in CD patients and suggest an etiological role of those top-five genes in the IFX response pathway.

## 1. Introduction

Crohn's disease (CD) is one of the clinical forms of inflammatory bowel disease (IBD) resulting from a defective regulation of mucosal immune responses to commensal microbiota in genetically susceptible individuals [1]. The last years have contemplated substantial progress in the identification of the genes involved in CD predisposition, boosted by the HapMap project and genome-wide association studies [2]. A better understanding of the biological pathways underlying CD pathogenesis will lead to the development of new therapeutic approaches that specifically target those pathways and will eventually allow personalized treatments. Therefore, an increasing need exists to predict the therapy most fitted to each patient.

Since 1998, when the US Food and Drug Administration approved infliximab (IFX) for treatment of moderate or severe CD that does not respond to a conservative treatment, monoclonal antibodies to tumor necrosis factor alpha (TNF-*α*) have become the hallmark treatment for refractory CD. Infliximab has proven to be effective for the treatment of both luminal [3] and fistulizing CD [4]. However, a lack of response or a partial response to IFX has been consistently observed and a growing need exists to identify biomarkers of response in order to achieve a more efficacious use of this expensive and potentially toxic therapy. Moreover, data from clinical trials of IFX suggest that high-risk patients and patients with active inflammation may benefit from earlier use of this drug [5]. Clinical parameters such as concurrent therapies, smoking habits, or previous surgery seem to account for only a small amount of the variance in response to anti-TNF therapies [6].

In a recent study by microarray analysis, pretreatment mucosal gene expression profiles predicted response to first IFX treatment in CD patients [7]. The study identified a 100% accurate predictive gene expression signature for response to IFX in Crohn's colitis; class prediction analysis allowed complete separation between responders and nonresponders through a panel of 5 top significant genes:* S100A8* and* S100A9, G0S2, TNFAIP6*, and* IL11*. This biological therapy has been also used for the treatment of other chronic inflammatory diseases such as rheumatoid arthritis (RA). RA and CD probably share a pathogenetic background, as they have been associated with overlapping susceptibility genes. Therefore, it is reasonable to expect that common genes would anticipate the response to this therapy in both conditions. Up to date, several studies used genome-wide expression analysis to identify expression signatures predicting response to anti-TNF treatment in RA patients, but results showed little overlap [8]. The identified expression profiles were often not consistent with each other, and different gene sets were reported to distinguish between responders and nonresponders. Therefore, despite the original promising results in CD, the expression signature could be considered a first step towards a predictive test. Provided corroboration from a genetic standpoint is achieved, the cluster of five genes with different expression in CD responders compared to nonresponders to IFX therapy would be a useful tool to classify patients. With this hypothesis, we aimed at investigating the reported expression profile by exploring the association of tagging variants in those top-five genes with the response to IFX in an independent Spanish cohort of CD patients. Once an association between polymorphisms within those genes and the response to IFX is found, a causal mechanism of IFX response would be envisaged and a simpler way to classify patients could be established, as it is much easier to perform genotyping of the involved genes than to check their levels of expression.

## 2. Material and Methods

### 2.1. Study Design and Patients

Overall, 350 unrelated white Spanish patients with active CD were consecutively recruited from 8 centers. Eligible patients were at least 18 years old, had an established diagnosis of CD, and had received at least the 3 induction doses of IFX (5 mg per kilogram) at weeks 0, 2, and 6. Diagnosis of CD was based on standard clinical, radiologic, endoscopic, and histological criteria [9]. IFX was administered to treat either moderate to severe active luminal CD or active fistulizing perianal CD.

Disease phenotype was determined following the Montreal Classification: age at diagnosis (A1: ≤16 years; A2: 17–40 years; A3: >40 years), anatomic location (L1: terminal ileum; L2: colon; L3; ileocolon; L4: upper gastrointestinal tract; and +L4: upper gastrointestinal modifier), and disease behavior (B1: inflammatory; B2: stricturing; B3: penetrating; and p: perianal modifier) [10].

Patients were classified as responders (remission or partial response) or nonresponders to IFX. The response to IFX was determined by a chronological review of the medical records and data were centrally monitored. The response to IFX in patients with luminal disease was evaluated by the Harvey-Bradshaw index (HBI) [11] at the beginning and 10 weeks after the first IFX dose. Partial response was defined as a decrease in the HBI of more than 3 points and absence of concomitant corticosteroids [12]. Remission was defined as a final HBI ≤4 and absence of concomitant corticosteroids [12]. In patients with perianal disease, response was evaluated at week 10 after the first IFX dose. Remission was defined as the complete closure of all fistulas and partial response as a reduction (≥50%) in the number of draining fistulas. Patients either receiving IFX for both luminal and fistulizing disease or achieving remission of any type that justified the maintenance of IFX treatment were considered responders. All patients who did not achieve partial response or remission after the three IFX induction doses were considered nonresponders.

### 2.2. Genotyping

In order to cover the highest variability within each gene showing altered expression, we chose single nucleotide polymorphisms (SNPs) by aggressive tagging from the HapMap B36 CEU population, which captured markers with *r* ^2^ > 0.8 (mean *r* ^2^ = 0.93) and a minor-allele frequency (MAF) >0.1. Genotyping of the Spanish samples was carried out with predesigned TaqMan Assays from Applied Biosystems (Applied Biosystems Inc., Foster City, CA, USA), in a 7900HT Fast Real-Time PCR system, under conditions recommended by the manufacturer. Genotyping call-rate success was over 95% for the SNPs in all groups of patients.

### 2.3. Data Analysis

**Figure 1:**
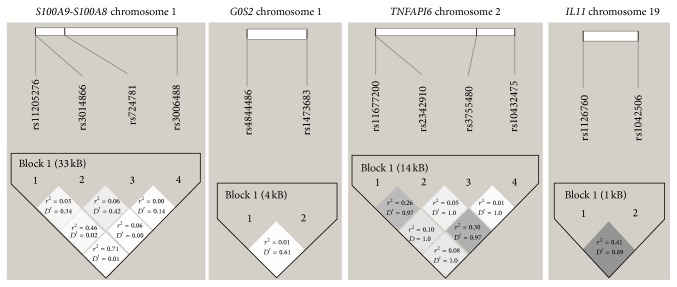
Linkage disequilibrium (*D*′ and *r* ^2^) between the studied SNPs located in the same genetic region:* S100A9*-*S100A8, G0S2, TNFAIP6*, and* IL11*.

Demographic and baseline characteristics were compared between responders and nonresponders by using the Mann-Whitney *U* test or the chi-square test, whether continuous or categorical variables were considered. The statistical analysis to compare allelic and genotypic distributions was performed using chi-square test or Fisher's exact test (when expected values were below 5). Odds ratios (ORs) were calculated and their 95% confidence intervals were estimated using the Cornfield method. Haplotypic frequencies were inferred with the expectation-maximization algorithm implemented in the Haploview 4.1 software. Linkage disequilibrium was measured by calculating two parameters: *r* ^2^ and *D*′ (Figure 1). Demographic characteristics were analyzed as potential confounding factors of the IFX response using logistic regression.

## 3. Results

Baseline characteristics of the Spanish patients, classified as responders or nonresponders to IFX therapy, are summarized in Table 1. A total of 285 (82%) patients were classified as responders and 62 (18%) as nonresponders. A statistically significant difference was observed between the years of evolution of disease in both groups.

Tagging polymorphisms in the five previously identified genes were genotyped in the two groups of CD patients, responders, and nonresponders to IFX therapy, and frequencies are shown in Table 2. The studied polymorphisms conformed to Hardy-Weinberg expectations. Although independently only rs11677200 in the* TNFAIP6* gene showed a statistically significant result, the aggressive tagging approach allowed a more thorough scrutiny of these genomic regions by analyzing the haplotypes conformed by those polymorphisms (haplotypic frequencies in responders and nonresponders are summarized in Table 3). In the region where the* S100A9, S100A12*, and* S100A8* genes map, the most frequent haplotype evidenced a significantly higher frequency in CD nonresponders than in responders to IFX (*P* = 0.051; OR (95% CI) = 1.54 (0.97–2.43)). Additionally, one haplotype in the* IL11* region showed a marginal significant association with the response to the anti-TNF treatment (*P* = 0.068; OR (95% CI) = 1.72 (0.91–3.21)), and trends for association could be observed for haplotypes within the other two chromosomal regions explored (*G0S2*: *P* = 0.15; OR (95% CI) = 2.92 (0.45–15.23) and* TNFAIP6*: *P* = 0.10; OR (95% CI) = 0.71 (0.46–1.09)).

The identification of an expression profile to predict response to IFX by Arijs and collaborators [7] was focused in colonic CD patients and therefore we evaluated the specific subgroups of Crohn's patients. Tables 4 and 5 summarize the results in ileal patients and colonic and ileocolonic patients, respectively. As shown, the haplotypes associated in the overall patients were now found significantly associated in the colonic subgroup in the* S100A9-S100A8* (Table 5(a), GCCA haplotype: *P* = 0.025; OR (95% CI) = 1.91 (1.05–3.48)), in the* G0S2* (Table 5(b), AT haplotype: *P* = 0.025; OR (95% CI) = 6.31 (0.82–47.89)) and in the* IL11* (Table 5(d), CT haplotype: *P* = 0.025; OR (95% CI) = 2.76 (1.39–5.44)) regions. In contrast, in the* TNFAIP6* gene, the most frequent CAGA haplotype only showed a trend for association in ileal patients (Table 4(c), CAGA haplotype: *P* = 0.1; OR (95% CI) = 0.57 (0.27–1.17)), while no difference was detected in colonic patients (Table 5(c)).

## 4. Discussion

Pharmacogenetics has emerged as a promising discipline which opens the possibility of a personalized medicine. However, research has been hampered mainly due to limitations that this kind of studies still shows. Recruitment of a high number of patients with a similar treatment is not an easy task, but difficulty increases because response criteria should be centrally monitored as they were in the present study. Moreover, the high success rate of some pharmacological therapies as IFX originates a low number of nonresponders, with the consequent decrease in the statistical power to detect differences. In our patients, induction therapy with IFX achieved a very good outcome with higher rates of response than those reported in controlled trials [3, 4], in agreement with a high response rate to the three induction doses of IFX previously reported in a multicenter study [13]. Moreover, a better response to IFX was observed in patients with an earlier treatment (Table 1), as already reported [14].

The hierarchical cluster analysis performed by Arijs and collaborators [7] identified a profile with five differentially expressed genes which was claimed to predict response to IFX in colonic CD patients with an overall accuracy of 100%. In that work, the authors validated a previously published gene expression signature regarding response to IFX in ulcerative colitis patients [15].* IL11* was the only overlapping gene between the two predictive top-five gene sets in both clinical forms of IBD. We aimed to investigate the causal implication of the described genes in the mechanism of IFX response through the association study of those genes in a cohort of Spanish CD patients. Moreover, this procedure might provide genetic markers of IFX response that would be easier to test.

In our independent cohort of CD patients, the tagging approach allowed us to explore a higher genetic variability within the chromosomal regions where the five genes map. Two of these five top genes are* S100A8* and* S100A9*; both encode members which belong to the S100 family of calcium-binding proteins and are located in a cluster on chromosome 1q21. Their expression is induced by proinflammatory cytokines such as IL-6 or TNF-*α* [16]. Calprotectin, the heterodimeric complex of S100A8 and S100A9, shows increased expression at an early step in the neoplastic transformation during colorectal carcinogenesis [17] and it is associated with disease activity in patients with IBD [18] and other inflammatory conditions as rheumatoid arthritis [19] or systemic lupus erythematosus [20]. Moreover, fecal calprotectin concentration is considered a useful surrogate marker for mucosal healing during TNF-*α* blocking therapy for IBD [21, 22]. In this genetic region, as mentioned, the most frequent haplotype was found significantly associated with response to IFX, mainly in colonic patients (22.7% responders versus 35.9% nonresponders, *P* = 0.025).

Another gene with reported downregulated expression in IFX responders is* IL11*. This interleukin is a member of the gp130 family of cytokines that stimulates T-cell dependent development of immunoglobulin-producing B cells, and that was tested as a therapy in CD [23]. In this case, association with borderline significance has been found involving an* IL11* haplotype with decreased frequency in CD responders to IFX. Moreover, a significant difference was evidenced when colonic CD patients were examined (11.5% responders versus 27.2% nonresponders, *P* = 0.0012).

One gene included in the predictive expression panel for IFX responsiveness is* TNFAIP6* (tumor necrosis factor, alpha-induced protein 6), which encodes a multifunctional protein with important roles in inflammation and tissue remodeling. It is upregulated in many inflammatory conditions as rheumatoid arthritis [24], a disease that also benefits from IFX therapy, and in colorectal cancer [25]. Another gene previously showing a differential expression profile predictive of response to IFX is* G0S2* (G0/G1 switch 2), involved in lymphocyte cell cycle regulation and found upregulated in rheumatoid arthritis and psoriasis [26, 27]. The haplotypes studied in these two genes evidenced trends for association with the response to IFX in the overall Spanish CD patients. Furthermore, only the trend for association observed in the* G0S2* gene could be significantly replicated in the colonic subgroup of patients (0.8% responders versus 5.2% nonresponders, *P* = 0.036).

The strategy followed in our study lends support to the reported gene expression profile predictive of anti-TNF therapy in an independent cohort of Spanish CD patients. Genetic studies stand out as approaches to define pathogenic pathways and ultimately the integration of genetic together with functional data promotes a clearer understanding of the mechanisms underlying therapeutic pathways.

## Figures and Tables

**Table 1:** Characteristics of Crohn's disease patients studied: both responders (*n* = 288) and nonresponders (*n* = 62) to infliximab.

	Responders		Nonresponders		*P* value
	*N*	%	*N*	%	
Age	39.6 ± 0.7		41.9 ± 1.5		0.10
Sex					
Male	135	47.4	26	41.9	0.44
Female	150	52.6	36	58.1	
Years of disease	10.2 ± 0.4		13.2 ± 1.1		0.0067
Age at diagnosis (A)					
A1	36	12.9	8	13.1	0.61
A2	203	72.5	47	77.1	
A3	41	14.6	6	9.8	
Location (L)					
L1	74	26.5	28	46.6	0.014^a^
L2	57	20.4	8	13.4	
L3	137	49.1	24	40.0	
L4	2	0.7	0	0	
L1 + L4	3	1.1	0	0	
L2 + L4	1	0.4	0	0	
L3 + L4	5	1.8	0	0	
Behavior (B)					
B1	79	28.2	19	31.7	0.56
B2	27	9.6	7	11.7	
B3	46	16.5	11	18.3	
B1p	70	25.0	8	13.3	
B2p	9	3.2	2	3.3	
B3p	49	17.5	13	21.7	

Data correspond at first IFX dose.

A1: ≤16 years; A2: 17–40 years; A3: >40 years. L1: terminal ileum; L2: colon; L3: ileocolon; L4: upper GI; L1 + L4: terminal ileum + upper GI; L2 + L4: colon + upper GI; L3 + L4: ileocolon + upper GI. B1: nonstricturing, nonpenetrating; B2: structuring; B3: penetrating; B1p: nonstricturing, nonpenetrating + perianal; B2p: structuring + perianal; B3p: penetrating + perianal.

^a^Excluding categories with L4.

**Table 2:** Genotype frequencies of the polymorphisms located in the genes studied in Crohn's disease patients: both responders and nonresponders to infliximab.

	Responders		Nonresponders	
	*N*	%	*N*	%
Gene *S100A9 *				
rs11205276				
GG	189	67	39	67
GC	88	31	18	31
CC	4	2	1	2
rs3014866				
CC	94	33	22	37
CT	129	46	30	51
TT	58	21	7	12
Gene *S100A12 *				
rs724781				
CC	128	46	27	47
CG	115	41	27	47
GG	37	13	4	6
Gene *S100A8 *				
rs3006488				
AA	235	83	47	78
AG	44	15	12	20
GG	5	2	1	2
Gene *G0S2 *				
rs4844486				
CC	145	52	33	54
CA	115	42	26	43
AA	18	6	2	3
rs1473683				
GG	269	97	54	95
GT	7	3	3	5
TT	0	0	0	0
GENE *TNFAIP6 *				
rs11677200^*^				
TT	79	29	26	45
TC	153	56	24	41
CC	42	15	8	14
rs2342910				
AA	138	52	30	57
AT	113	42	18	34
TT	16	6	5	9
rs3755480				
GG	218	78	44	72
GA	57	20	15	25
AA	6	2	2	3
rs10432475				
AA	224	80	44	77
AG	54	19	12	21
GG	2	1	1	2
Gene *IL11 *				
rs1126760				
TT	165	60	28	52
TC	94	34	24	44
CC	16	6	2	4
rs1042506				
TT	195	76	37	77
TG	52	20	11	23
GG	9	4	0	0

^*^CC genotype, *P* = 0.017; OR (95% CI) = 0.5 (0.27–0.93).

**(a):** 

Haplotype	Responders (%)	Nonresponders (%)	*P* value
GCCA	22.8	31.4	0.05
GTCA	24.6	18.4	0.16
GCGA	21.5	19.7	0.63
CTCA	9.3	10.9	0.57
GTGA	4.9	1.9	0.11
CCCA	4.1	3.4	0.73
GCCG	3.8	4.4	0.77
GTGG	2.4	4.4	0.28
CCGA	2.1	1.8	0.77
GCGG	1.5	1.7	0.92
GTCG	1.3	1.0	0.72
CTGA	1.1	0.7	0.83

**(b):** 

Haplotype	Responders (%)	Nonresponders (%)	*P* value
CG	72.4	74.3	0.61
AG	26.3	23.0	0.43
AT	0.8	2.4	0.12

**(c):** 

Haplotype	Responders (%)	Nonresponders (%)	*P* value
CAGA	43.0	34.8	0.10
TAGA	17.4	21.6	0.24
TTGA	16.8	15.3	0.68
TAAA	12.3	16.1	0.24
TTGG	10.2	12.1	0.60

**(d):** 

Haplotype	Responders (%)	Nonresponders (%)	*P* value
TT	76.5	72.2	0.38
CG	12.8	10.0	0.50
CT	10.0	15.7	0.07
TG	0.7	2.0	0.25

**(a):** 

Haplotype	Responders (%)	Nonresponders (%)	*P* value
GTCA	36	23.7	0.16
GCCA	22.8	28.4	0.48
GCGA	12.2	19.4	0.16
CTCA	10.3	12.6	0.57
GTGG	2.6	5.5	0.39
GTGA	3.7	2.5	0.68
CCCA	3.3	3.3	1.00
CCGA	3.5	2.6	1.00
GCGG	3	1.4	1.00
GCCG	1.6	0.4	1.00

**(b):** 

Haplotype	Responders (%)	Nonresponders (%)	*P* value
CG	71.5	75.8	0.54
AG	25.7	24	0.82
CT	2.1	0.1	0.57

**(c):** 

Haplotype	Responders (%)	Nonresponders (%)	*P* value
CAGA	39.4	27.1	0.14
TAGA	20.2	24.4	0.56
TAAA	14.9	24.1	0.13
TTGA	16.1	10.7	0.37
TTGG	9.5	13.7	0.47

**(d):** 

Haplotype	Responders (%)	Nonresponders (%)	*P* value
TT	77.6	83	0.40
CG	16	13.5	0.74
CT	5.6	3.4	1.00

**(a):** 

Haplotype	Responders (%)	Nonresponders (%)	*P* value
GCGA	25.1	21.5	0.56
GCCA	22.7	35.9	0.025
GTCA	20.7	11.6	0.10
CTCA	8.8	10.8	0.52
GTGA	5.2	1.8	0.33
GCCG	4.1	7.2	0.33
CCCA	4.2	3.5	1.00
GTGG	2.4	1.2	1.00
GTCG	2.1	2.3	1.00
CCGA	1.6	1	1.00
GCGG	1.2	1.6	0.58
CTGA	1.1	0.6	0.52
CCCG	0.8	1.1	0.44

**(b):** 

Haplotype	Responders (%)	Nonresponders (%)	*P* value
CG	72.1	71.7	0.94
AG	27.1	23.2	0.54
AT	0.8	5.2	0.036

**(c):** 

Haplotype	Responders (%)	Nonresponders (%)	*P* value
CAGA	44.5	44.1	0.87
TAGA	17.2	19.4	0.61
TTGA	15.7	16.6	0.86
TAAA	11.9	8.3	0.43
TTGG	10.3	11.6	0.74

**(d):** 

Haplotype	Responders (%)	Nonresponders (%)	*P* value
TT	76	62.8	0.040
CT	11.5	27.2	0.0012
CG	11.7	6.1	0.17
TG	0.8	3.9	0.12
